# Germline Genetic Testing in Breast and Gynecologic Cancers: Evaluating Age at Diagnosis as a Determinant

**DOI:** 10.3390/cancers18101541

**Published:** 2026-05-10

**Authors:** Eirini Papadopoulou, Georgios N. Tsaousis, Romina Alevizou, Dimitrios Alexandrou, Theodoros Argyriou, Anna Giannopoulou, Markos Thanos, Sofia Kakoulaki, Christos Kalyvopoulos, Maria Kanara, Christos Kanistras, Nikolaos Katsiakis, Anastasios Katsourakis, Dimitrios Kokkonis, Theodoros Kontoulis, Ioanna Konstantiadou, Dimitrios Tryfonopoulos, Sofia Karageorgopoulou, Anna Koumarianou, Dimitrios Ziogas, Stavros Bikos, Effrosyni Bompou, Georgios Boutsikos, Varvara Pantelidou, Aikaterini Savvidou, Vasileios Sakellariou, Maria Matiatou, Panagiotis Karathanasis, Maroulio Stathoulopoulou, Vassileios Venizelos

**Affiliations:** 1 Genekor Medical S.A., 15344 Athens, Greece; gtsaousis@genekor.com; 2 2nd Breast Clinic, IASO General Clinic, 15123 Athens, Greece; alevizouromina@gmail.com; 3 Papageorgiou General Hospital, 56403 Thessaloniki, Greece; dimalexthess@gmail.com; 4 St. Luke’s Hospital, 55236 Thessaloniki, Greece; argyriou@dr.com (T.A.); theo.kontoulis@gmail.com (T.K.); s.bikos@yahoo.gr (S.B.); savvidouaik@gmail.com (A.S.); vasakelariou@gmail.com (V.S.); 5 Henry Dunant Hospital, 11526 Athens, Greece; annagianno@yahoo.gr; 6 Apolloneio-Theotokos Clinic, 54646 Thessaloniki, Greece; m.thanos@email.de; 7 Breast Surgery Clinic, 71201 Heraklion, Greece; sofiakako@yahoo.gr; 8 Agios Savvas Anticancer Hospital, 11522 Athens, Greece; chris.kalivopoulos@gmail.com; 9 Trikala General Hospital, 42131 Trikala, Greece; makanara1@hotmail.com; 10 Genesis, 57001 Thessaloniki, Greece; chkanistras@yahoo.com; 11 Olympion General Clinic, 26443 Patras, Greece; nikos_katsiakis@windowslive.com; 12 Agios Dimitrios General Hospital, 54634 Thessaloniki, Greece; tasoskatsourakis@hotmail.com; 13 Interbalkan Medical Center, 57001 Thessaloniki, Greece; kokkonis.surgery@gmail.com; 14 Thessalia General Clinic, 41334 Larissa, Greece; kostasioanna@yahoo.gr; 15 Second Department of Medical Oncology, Agios Savvas Anticancer Hospital, 11522 Athens, Greece; tryfonopoulos@hotmail.com; 16 Third Department of Medical Oncology, IASO Clinic, 15123 Athens, Greece; skarageorgopoulou@iaso.gr; 17 Attikon University Hospital, National and Kapodistrian University of Athens, 12462 Athens, Greece; akoumari@yahoo.com; 18 General Hospital of Athens LAIKO, 11527 Athens, Greece; ziogasdc@med.uoa.gr; 19 University General Hospital of Larissa, 41334 Larissa, Greece; feybobou@hotmail.com; 20 IASO Thessalias, 41500 Larissa, Greece; gboutsikos@iaso.gr; 21 424 General Military Hospital of Thessaloniki, 56429 Thessaloniki, Greece; bpantelidoy79@gmail.com; 22 ΕUSOMA—Certified Multidisciplinary Breast Center, Metropolitan Hospital, 18547 Piraeus, Greece; mmatiatou@metropolitan-hospital.gr (M.M.); pkarathanasis@yahoo.gr (P.K.); maroulio.stath@gmail.com (M.S.); bvenizelos@metropolitan-hospital.gr (V.V.)

**Keywords:** hereditary cancer, genetic testing, multigene panel, age at diagnosis, BRCA1/2, breast cancer, ovarian cancer

## Abstract

Background: Breast cancer and gynecological malignancies, including cervical, ovarian, and endometrial cancers, remain leading causes of cancer incidence and mortality among women worldwide. This study investigated hereditary predisposition rates in women diagnosed with breast or gynecological cancer, focusing on the effect of age on germline pathogenic/likely pathogenic (P/LP) variant detection. We sought to determine whether younger age at diagnosis should be used as a criterion for patient selection for genetic testing. Methods: A total of 9084 consecutive females with breast cancer or gynecological tumors underwent germline NGS-based genetic testing (52 cancer-relevant genes) at Genekor laboratory from 2020–2026. Multivariable logistic regression evaluated factors associated with P/LP variant detection, adjusting for tumor type and family history. Results: Overall P/LP prevalence was approximately 20% (one in five patients), with tumor-specific rates of 19.24% in breast cancer, 27.59% in ovarian cancer, and 26.67% in endometrial cancer. P/LP prevalence declined significantly with age from 24.37% in patients <40 years to 15.90% in those ≥70 years, while Variants of Uncertain Significance (VUS) remained stable (40–43%). P/LP patients had earlier diagnosis (median 45 vs. 46 years, *p* < 0.001), driven predominantly by high-risk genes (13.87% in <40 y vs. 7.11% in ≥70 y). *BRCA1* showed stronger age enrichment than *BRCA2* (8.14% vs. 3.16% in <40 y; median diagnosis 43 vs. 45 years). Age remained independently associated with P/LP detection in multivariable analysis, with an 18% reduction in odds per 10-year increase for any P/LP (OR 0.82, 95% CI 0.78–0.86) and a stronger 28% reduction for high-risk variants (OR 0.72, 95% CI 0.67–0.78). Family history also independently predicted P/LP detection (OR 1.40, 95% CI 1.19–1.66). Conclusions: Although derived from a referral-based (and thus selected) population, these findings show that while younger patients have a higher prevalence of high-risk P/LP variants, clinically actionable findings are present across all age groups, including those ≥70 years. These results suggest that reliance on age alone to determine eligibility for genetic testing may be insufficient. Broadening access to testing beyond strict age-based criteria could improve the identification of hereditary cancer risk and inform patient management, although further evaluation in less selected populations is warranted.

## Simple Summary

This study examined how age affects pathogenic/likely pathogenic (P/LP) variant detection in breast and gynecological cancer patients with hereditary susceptibility. We investigated whether younger age at diagnosis should be utilized for selecting genetic testing patients. Overall P/LP prevalence was approximately 20% (one in five patients), P/LP prevalence declined significantly with age from 24.37% in patients <40 years to 15.90% in those ≥70 years, while VUS remained stable (40–43%). P/LP patients had earlier diagnosis (median 45 vs. 46 years, *p* < 0.001), driven predominantly by high-risk genes. Age remained independently associated with P/LP detection in multivariable analysis, with an 18% reduction in odds per 10-year increase for any P/LP and a stronger 28% reduction for high-risk variants. Family history also independently predicted P/LP detection. These findings indicate that focusing on age to determine eligibility for genetic testing is insufficient. Expanding access to testing for individuals regardless of age at diagnosis may improve the identification of genetic cancer risk and optimize patient management.

## 1. Introduction

Breast cancer and gynecological malignancies, including cervical, ovarian, and endometrial cancers, continue to be among the most prevalent causes of cancer incidence and mortality in women worldwide. Among these, breast cancer is the most frequently diagnosed cancer in females, with approximately 2.30 million new cases reported globally in 2022, accounting for approximately 670,000 deaths worldwide [1]. Ovarian cancer is the third most prevalent gynecological tumor type, and the one exhibiting the highest mortality rate, mainly since diagnosis usually occurs at metastatic stages [1,2]. Consequently, efforts have focused on earlier diagnosis to reduce the burden of these malignancies. These strategies include population screening and early-detection programs, as well as hereditary germline testing for cancer predisposition in selected individuals. While population screening and early-detection programs aim to prevent disease onset or identify disease at an earlier stage, germline genetic testing in patients already diagnosed with cancer provides information on hereditary predisposition, family risk assessment, and clinical management, and may also inform treatment strategy [3].

Familial and hereditary factors account for a substantial proportion of breast and ovarian cancers. Pathogenic germline variants in high-penetrance genes are identified in roughly 5–10% of breast cancers and about 25% of ovarian cancers [4]. While a significant proportion of hereditary breast–ovarian cancer is attributable to *BRCA1* and *BRCA2* alterations, growing evidence suggests that additional high- and moderate-risk genes, such as *PALB2*, *RAD51C*, *RAD51D*, *BRIP1*, *ATM*, *CHECK2*, and others, have a substantial contribution to hereditary breast and ovarian cancer risk [5]. Therefore, multigene panel testing has been shown to substantially increase diagnostic yield in patients at hereditary risk and to inform surveillance, prevention, and family testing decisions, while also introducing additional complexity to risk interpretation, particularly for moderate- and low-penetrance genes and variants of uncertain significance (VUS) [4,6]. Furthermore, Lynch syndrome is responsible for a substantial hereditary proportion of endometrial malignancies and certain ovarian cancers, whereas the monogenic hereditary contribution of cervical cancer is undefined or minimal [7,8]. Genetic counseling and testing in women at risk is imperative for the development of personalized surveillance and preventive strategies to decrease the morbidity and mortality associated with these hereditary cancer syndromes, as well as for the identification of cancer risk within the family [5,9].

In recent years, the widespread use and development of advanced molecular techniques, spectrum of cancer-associated genes, clarifying their role in cancer predisposition and the appropriate management of patients based on their genetic background [10,11]. Consequently, the analysis of hereditary cancer gene panels by next-generation sequencing (NGS) is crucial not only before but also after a cancer diagnosis. This analysis can provide valuable information regarding the disease course, treatment, and the likelihood of a second malignancy, depending on the affected gene. Importantly, such a test is more informative when performed at the time of cancer diagnosis [10,12,13]. This is particularly useful in breast cancer since the knowledge of genetic analysis results may influence surgical planning and inform the consideration of more extensive procedures, including risk-reducing bilateral mastectomy [9,14,15]. Such decisions, however, are individualized and rely on comprehensive risk assessment that integrates personal and family cancer history, gene-specific penetrance, and patient preference, in line with current clinical guidelines [9].

Nevertheless, a primary concern is whether a cancer diagnosis alone is sufficient for hereditary testing. With the increasing use of targeted therapies informed by genes included in hereditary cancer panels, the clinical importance of germline testing has progressively expanded. *BRCA1/2* testing, in particular, is essential for guiding decisions about PARP inhibitors in breast and ovarian cancer. Some consensus statements, such as the American Society of Breast Surgeons’ recommendation, have proposed extending genetic testing to all women with a current or prior breast cancer diagnosis [16]. Nevertheless, this broader approach has not been universally adopted by major international guidelines, which continue to apply specific age, tumor, and family-history-based eligibility criteria [17,18,19]. For ovarian cancer, though, guidelines recommend genetic testing at diagnosis due to the high incidence of *BRCA1/2* alterations and the treatment implications in positive cases [2,20]. At the same time, current evidence does not support germline analysis at diagnosis for other gynecological malignancies. However, if MMR deficiency is detected, it is recommended that the MMR genes be analyzed in the context of *MLH1*-unmethylated tumors [21].

Our research focused on the hereditary predisposition rates of women diagnosed with breast or gynecological cancer, in accordance with the most recent evidence. Furthermore, the effect of age on the likelihood of a positive result was investigated in a group of female cancer patients referred for genetic testing with known age at diagnosis. In this way, we sought to evaluate the association between age at diagnosis and P/LP detection within a referred population, and to explore whether age alone adequately captures hereditary risk in this context.

## 2. Materials and Methods

### 2.1. Patients

In the current study, 9084 consecutive females with breast cancer or a gynecological tumor were referred to Genekor’s laboratory for NGS-based genetic testing during the period from 2020 to 2026. Before undergoing molecular genetic testing, they all provided informed consent, granting permission for the anonymized use of their genetic data in scientific research and the potential publication of research findings. The study protocol was reviewed and approved by the Scientific and Ethics Committee of the Hellenic Society of Breast Surgeons (EXEM) (Protocol No: 00113; approval date: 2 April 2026).

Family history data were available for the majority of patients and were included in multivariable analyses, although detailed pedigree data were not accessible. All patients were referred for testing by the treating physician due to suspicion of hereditary cancer based on the age of diagnosis and/or the family history of cancers. A family history of any cancer was reported in 7622 (83.96%) patients, including 3788 (41.69%) who had a family history of the same tumor type, while 1456 (16.04%) had no family history of cancer, and 6 (0.07%) had an unknown status. Among these, 4482 Breast and ovarian cancer, the patients’ referral for genetic testing was based on criteria established by the main public health insurance body in Greece (EOPYY), for reimbursement of genetic testing. Eligibility criteria were: (i) ovarian cancer, regardless of age or family history; (ii) breast cancer diagnosed before the age of 45 years; (iii) breast cancer in patients with at least two first- or second-degree relatives with a confirmed diagnosis of breast, ovarian, pancreatic, or prostate cancer (Gleason score ≥ 7). The present analysis focused primarily on the contribution of age at diagnosis to the diagnostic yield of genetic testing, while accounting for tumor type and family history where available.

### 2.2. Statistical Analysis

All statistical analyses were performed in 9084 patients with available age at diagnosis. Categorical variables were summarized as counts and percentages, and age at diagnosis was summarized using medians. Comparisons of age at diagnosis between two independent groups were performed using the two-sided Mann–Whitney U test, given the non-normal distribution of age-related variables [22]. Comparisons of age at diagnosis between two independent groups were performed using the two-sided Mann–Whitney U test. Age distributions were assessed by visual inspection (histograms and Q–Q plots) and found to be skewed; therefore, non-parametric methods were selected to provide robust comparisons based on rank distributions and medians, which are less influenced by skewness and potential outliers. This approach was used for the primary comparisons of P/LP-positive versus mutation-negative patients; VUS versus mutation-negative patients; multi-gene versus single-gene P/LP carriers; and truncating versus non-truncating variant groups within the same gene [23].

To assess the relationship between age and hereditary test result category, patients were stratified into age groups (<40, 40–49, 50–59, 60–69, and ≥70 years), and distributions were compared using chi-square tests of independence, with pairwise post-hoc comparisons where appropriate. Pairwise comparisons of P/LP detection rates across age groups, performed separately for high-penetrance, intermediate, low/unclassified, and any P/LP categories, were computed using two-sided Fisher’s exact tests and adjusted for multiple testing within each variant-category family using the Benjamini–Hochberg false discovery rate (FDR) method.

To evaluate the association between age at diagnosis and the likelihood of detecting a pathogenic or likely pathogenic (P/LP) variant, multivariable logistic regression models were constructed. The primary outcome was the presence of at least one P/LP variant (yes/no). In a secondary analysis, the outcome was restricted to high-risk P/LP variants, defined as variants in established high-penetrance cancer predisposition genes (including *BRCA1*, *BRCA2*, *PALB2*, *TP53*, *PTEN*, *EPCAM*, *MLH1*, *MSH2*, *MSH6*, and *PMS2*). Age at diagnosis was modeled as a continuous variable. For interpretability, effect estimates are presented per 10-year increase in age. To assess potential non-linearity, age was also modeled categorically (<40, 40–49, 50–59, 60–69, ≥70 years; reference <40 years), and the categorical and continuous models were compared using likelihood ratio tests.

The primary model included the following covariates: age at diagnosis, tumor type (breast, ovarian, endometrial), family history of cancer (yes/no), and family history of the same cancer (yes/no). A secondary model, including only age and tumor type, was also evaluated as a sensitivity analysis. Odds ratios (ORs) with 95% confidence intervals (CIs) were reported. All tests were two-sided, and *p* < 0.05 was considered statistically significant.

### 2.3. Genetic Testing

Blood samples were collected from all patients and were analyzed for hereditary cancer predisposition using a custom hybrid capture-based NGS approach targeting 52 cancer-relevant genes. Sample preparation and hybrid capture were performed using the SeqCap EZ Choice Library and NimbleGen SeqCap EZ Choice kit (ROCHE diagnostics, Pleasanton, CA, USA), according to the manufacturer’s instructions as previously described [24]. Τhe assay targets all coding regions of the indicated transcripts and 20 base pairs of flanking intronic sequences and 25 base pairs of flanking intronic sequences for *BRCA1* and *BRCA2* genes.

Sequencing of the prepared samples was carried out utilizing either DNBG400 or DNB-T7 platform technology provided by MGI Tech Co., Ltd. (Beishan Industrial Zone, Shenzhen, China). All targeted regions within exons were sequenced with ≥20x depth. The generated sequencing data underwent analysis using the SeqNext version 4.4.0 software suite developed by JSI Medical Systems GmbH (Ettenheim, Germany). This software facilitated the identification and interpretation of sequence alterations within the context of clinically relevant transcripts.

The presence of large genomic rearrangements (LGRs) for all genes was investigated using the commercial computational algorithm SeqPilot Version 4.4 Build 505 (JSI Medical System) with the aim of detecting exon and gene-level deletions or duplications. In addition, the computational algorithm panelcn. MOPS was also used in the *BRCA1* and *BRCA2* genes. The presence of LGRs was verified by using the MLPA method (Multiplex Ligation-dependent Probe Amplification, MRC Holland B.V., Amsterdam, The Netherlands) [25,26].

Genes were categorized into high-, intermediate-, and low/unclassified- risk (penetrance) groups based on current literature and clinical guidelines [9,27,28,29,30]. High-risk genes included established high-penetrance cancer predisposition genes with well-defined clinical management implications, encompassing both hereditary breast and ovarian cancer genes (*BRCA1*, *BRCA2*, *PALB2*, *TP53*, *PTEN*) and Lynch syndrome mismatch repair genes (*EPCAM*, *MLH1*, *MSH2*, *MSH6*, *PMS2*), which are most relevant to endometrial and ovarian cancer predisposition. Although these genes have distinct tumor-type associations, they were grouped together as high-penetrance findings to allow a unified estimation of the age effect across the full gene panel. Intermediate-risk genes included those with moderate and consistently reported associations with cancer risk (e.g., *CHEK2* truncating variants, *ATM*, *BRIP1*, *RAD51C/D*, *BARD1*). Genes with limited, context-dependent, or uncertain associations with breast and gynecologic cancer risk—including *MUTYH*, *APC*, *CHEK2* missense variants, *RAD50*, *FANCA*, and others—were categorized as low/unclassified.

## 3. Results

### 3.1. Cohort Characteristics

Breast cancer was the predominant testing indication (88.8%), followed by ovarian cancers (9.5%), endometrial cancers (1.5%), and cervical cancer (0.2%). Median age at diagnosis differed across groups, with the breast cancer cohort showing a younger median age (45 years) than the ovarian (57 years) and endometrial cancer (55 years) groups; the cervical subgroup had a median age of 49 years (Table 1).

### 3.2. Genetic Testing Analysis Results

Among the 9084 patients included in this referral-based cohort, 20.09% (1825/9084) had at least one pathogenic/likely pathogenic (P/LP) finding (Supplementary Table S1). This proportion reflects the detection yield within patients referred for hereditary cancer testing, including a substantial subset tested under public health insurance reimbursement criteria, rather than a population-level prevalence estimate in unselected patients with breast or gynecologic cancer. These patients contributed a total of 1966 distinct P/LP findings. Most P/LP-positive patients had a single finding, whereas 136/1825 (7.45%) harbored more than one distinct P/LP finding, including 132 with alterations in different genes and 4 with multiple P/LP findings within the same gene. Among P/LP findings, truncating variants—defined as frameshift, nonsense, splicing, start-loss, and copy-number variants—predominated (73.24%), whereas non-truncating variants (missense and in-frame insertions/deletions) were less common (Supplementary Table S2). Notably, copy number variants (CNVs), including exon-level deletions and duplications, were not rare. They were present in 1.23% of the overall tested cohort and accounted for 6.14% of P/LP-positive patients. These findings indicate that a meaningful fraction of actionable hereditary findings would be missed by NGS approaches lacking adequate CNV detection. CNVs were observed predominantly in clinically important predisposition genes, especially *BRCA1* (65 carriers) and *CHEK2* (20 carriers), with additional CNV-positive cases involving *BRCA2*, *PMS2*, *FANCA*, *PALB2*, *BARD1*, *RAD51C*, *CDH1*, *ATM*, *BRIP1*, *MLH1*, *MSH2*, and *MUTYH*. These observations support the inclusion of CNV detection in NGS-based hereditary cancer testing.

**Figure 1:**
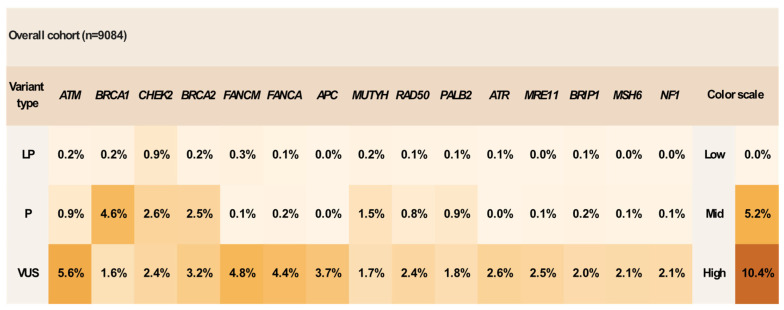
Genetic variant distribution heatmap of the most altered genes in the entire cohort. Each cell represents the percentage of patients harboring a specific variant type in the corresponding gene.

**Figure 2:**
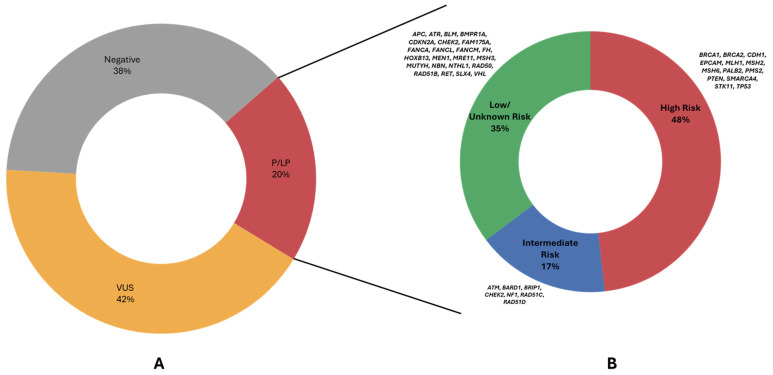
Charts illustrating the categorization of patients based on the most clinically significant variant detected in the overall referral-based cohort (**A**) and the distribution of gene risk category among patients with P/LP findings (**B**).

The most frequently altered genes (identification of P/LP variants) at patient level were *BRCA1* (439 patients, 4.83% of the cohort; 24.05% of P/LP-positive patients), *CHEK2* (315, 3.47%; 17.26%), *BRCA2* (242, 2.66%; 13.26%), *MUTYH* (149, 1.64%; 8.16%), *ATM* (98, 1.08%; 5.37%), *PALB2* (97, 1.07%; 5.32%), *RAD50* (79, 0.87%; 4.33%), and *RAD51C* (49, 0.54%; 2.68%) (Figure 1). These findings indicate that hereditary cancer susceptibility in this population extends well beyond the classic *BRCA1/2* paradigm, with a substantial contribution from additional predisposition genes detectable only through broad-panel testing. High-penetrance genes represented an important component of the positive cohort and included *BRCA1*, *BRCA2*, *PALB2*, *TP53*, *PTEN*, *EPCAM*, *MLH1*, *MSH2*, *MSH6*, and *PMS2*. Additionally, a substantial proportion of positive findings also involved intermediate-risk genes such as *CHEK2* (truncating variants), *ATM*, *BRIP1*, *RAD51C*, *RAD51D*, *BARD1*, and *NF1*, as well as lower/unclassified genes including *RAD50*, *FANCL*, *NBN*, *FANCM*, *RAD51B*, ATR, *FANCA*, and *MSH3* (Figure 2). *CHEK2* and *APC* missense variants were considered as low penetrance. In addition, alterations in genes not classically associated with hereditary breast or gynecologic cancer predisposition in the context of the presenting phenotype, such as *CDKN2A*, *RET*, and *VHL*, were considered incidental findings and categorized as unclassified [28,31,32,33]. For genes with broader or phenotype-dependent tumor-spectrum associations, including *APC*, *MUTYH*, and *NTHL1*, findings were not considered core phenotype-defining alterations in the present analysis but were retained for descriptive reporting [28,34].

**Figure 3:**
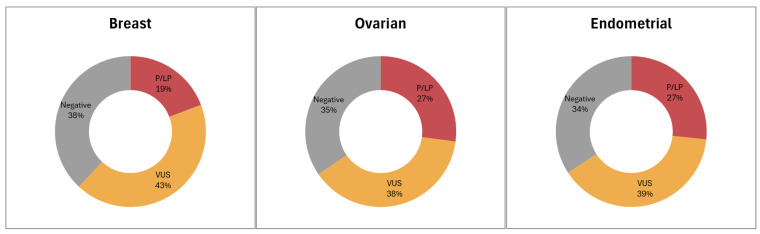
Charts illustrating the categorization of patients based on the most clinically significant variant detected through genetic testing results across the major tumor types.

The prevalence of P/LP findings differed significantly across tumor groups (χ^2^ *p* = 5.57 × 10^−8^). P/LP variants were identified in 19.24% of breast cancer patients, 27.59% of ovarian cancer patients, 26.67% of endometrial cancer patients (Figure 3). Thus, although breast cancer accounted for the vast majority of tests numerically, the proportional yield of actionable findings was higher in the major gynecologic cancer groups. In the cervical subgroup, P/LP findings were observed in 22.73% of cases, although this estimate should be interpreted with extreme caution given the very small sample size (*n* = 22), and we consider these data exploratory rather than definitive. Additionally, although the positivity rate appears higher in patients with gynecologic tumors, it should be noted that these patients differed from breast cancer patients in age distribution and in eligibility criteria for referral, with ovarian cancer patients eligible for genetic testing at any age under the applicable reimbursement framework.

Penetrance profile of P/LP findings varied across tumor types, with ovarian cancer displaying the highest rate of high-penetrance findings (18.28%), while in breast and endometrial cancer, the high-penetrance rate was 8.68% and 12.41%, respectively. Intermediate penetrance findings occurred in similar frequencies across breast and ovarian cancers (3.29% and 3.79%, respectively) but were less common in endometrial cancer (1.46%). Additionally, the prevalence of low penetrance gene findings for the 3 major tumor types, breast, ovarian, and endometrial, was 7.27%, 5.06%, 10.95%, respectively. In cervical cancer, high- and intermediate-penetrance findings occur at similar frequencies and (9.09% each), with low-penetrance findings observed less often (4.55%), although interpretation is limited by the small sample size.

Overall, these data suggest that in the tested cohort, ovarian cancer is driven more strongly by high-penetrance predisposition genes, whereas breast and endometrial cancers display a more heterogeneous penetrance spectrum. In ovarian cancers, the P/LP spectrum remained strongly *BRCA*-centered, whereas endometrial cancers were enriched for mismatch repair genes. However, this pattern reflects findings in a cohort enriched for hereditary risk through clinician-directed referral and reimbursement eligibility, and may not be representative of the gene contribution observed in unselected, population-based ovarian cancer cohorts, where moderate-penetrance genes such as *BRIP1*, *RAD51C*, and *RAD51D* contribute a meaningful fraction of pathogenic variants [29].

**Figure 4:**
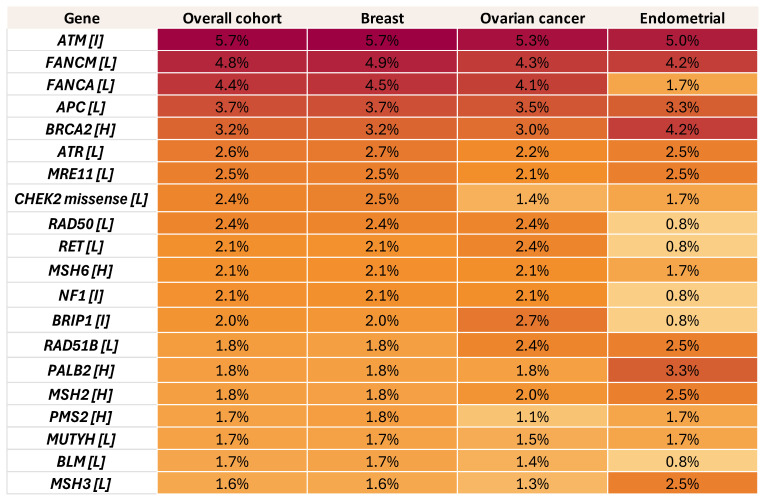
Heatmap illustrating the distribution (%) of VUS across genes in the overall cohort and within breast, ovarian, and endometrial cancer subgroups. Each cell represents the percentage of patients harboring a VUS in the corresponding gene and tumor type. Color intensity reflects variant frequency, with darker shades indicating higher prevalence. Genes are categorized according to their associated cancer risk level: high-risk (H), intermediate-risk (I), and low-risk (L).

A substantial proportion of VUS were also identified in our cohort. 4788 patients (52.71%) carried at least one VUS in at least one gene; however, among these patients, some patients also carried P/LP findings, therefore they were categorized in the P/LP category, and VUS results were not of clinical relevance. At a patient level, 3834/9084 patients (42.21%) had a genetic result categorized as VUS, meaning that a VUS was the highest tier variant detected (Figure 4). VUS results were most common in breast cancer at 42.71% (3440/8055), followed by endometrial at 40.15%, ovarian/fallopian/peritoneal at 38.28% (333/870), and cervical cancer at 27.27% (6/22). Among these, 1396 patients (15.37% of the whole cohort; 29.16% of VUS carriers) had at least one VUS in a high-penetrance gene, most commonly *BRCA2* (288 patients), *MSH6* (189), *PALB2* (164), *MSH2* (162), *PMS2* (157), and *BRCA1* (145).

### 3.3. Prevalence of Genetic Analysis Findings by Diagnostic Age

In addition, the prevalence of P/LP variants was examined in relation to the age of diagnosis. Patients with any P/LP finding had earlier onset of the disease compared to mutation-negative patients overall (median 45 vs. 46 years; Mann–Whitney *p* = 6.18 × 10^−8^), and this difference was most strongly driven by high-penetrance genes (overall high-tier vs. negative: median 45 vs. 46 years; *p* = 7.28 × 10^−9^). Although statistically significant, these median-level differences are small in absolute terms and may reflect the high statistical power of the cohort rather than a clinically meaningful shift in age at presentation. Comparing patients with any P/LP finding (n = 1825) and those with a VUS-only result (n = 3834) both had a median diagnosis age of 45 years; however, their age distributions differed significantly (Mann–Whitney *p* = 1.31 × 10^−4^).

**Figure 5:**
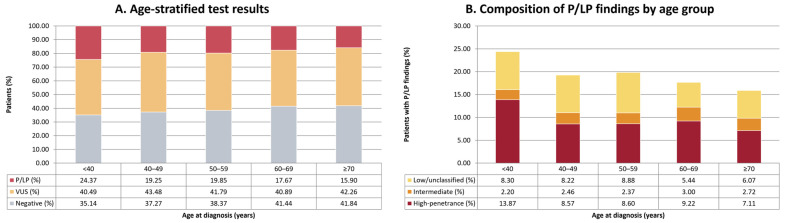
Age-related patterns of hereditary findings in the referral-based cohort. (**A**) Stacked bar plot showing the proportions of negative, VUS, and P/LP results across age groups at diagnosis. (**B**) Stacked bar plot showing the relative contribution of high-penetrance, intermediate, and low/unclassified categories to the total P/LP rate in each age group. These data illustrate that the observed decline in overall hereditary positivity with age is primarily attributable to a reduction in high-penetrance findings.

Age-group analysis supported a clear distinction between P/LP findings and variants of uncertain significance (VUS) (Table 2, Figure 5A). Across the age-available cohort (*n* = 9084), the distribution of hereditary testing results differed significantly by age group (χ^2^(8) = 37.77, *p* = 8.31 × 10^−6^). The prevalence of P/LP findings was higher in younger patients and declined with age, from 24.37% in those aged <40 years to 15.90% in those aged ≥70 years, corresponding to an absolute difference of 8.47 percentage points, indicating a moderate age-related gradient. In contrast, VUS results remained relatively stable across the age spectrum, ranging from 40.49% to 43.48% across age bands, whereas negative results increased modestly with age. In pairwise age-band comparisons, the distribution of VUS and negative results did not differ significantly (*p* = 0.201), whereas P/LP findings differed significantly from both negative results (*p* = 2.53 × 10^−6^) and VUS results (*p* = 1.51 × 10^−4^). These findings indicate that, unlike P/LP findings, both negative and VUS results do not show a meaningful age-related enrichment pattern.

Across the overall cohort, the age-related signal was driven mainly by high-penetrance findings, which were most frequent in younger patients: 13.87% in those aged <40 years versus 7.11% in those aged ≥70 years, corresponding to an absolute difference of 6.76 percentage points (OR 2.10, FDR-adjusted q = 1.20 × 10^−4^). In contrast, no significant differences were observed among groups aged ≥40 years after FDR correction. A consistent pattern was observed for any P/LP finding (<40 vs. ≥70: 24.37% vs. 15.90%; OR 1.70, q = 2.18 × 10^−4^). Conversely, intermediate-tier findings showed no significant variation across age groups (omnibus *p* = 0.77; all FDR q ≥ 0.93), and low/unclassified-tier findings showed only modest, non-monotonic differences involving the 60–69 group (Table 3, Figure 5B).

**Figure 6:**
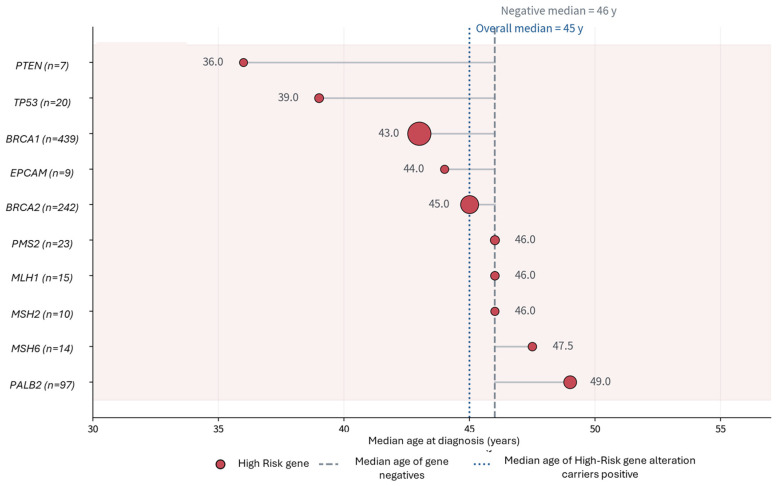
Median age at diagnosis according to the high-risk (penetrance) gene with a detected P/LP variant in the cohort. Bubble size reflects the number of unique patients with a P/LP finding in each high-risk gene.

Moreover, among high-risk P/LP variants, *TP53*, *PTEN*, and *BRCA1* are associated with the youngest median ages at diagnosis, whereas *PALB2* and several mismatch-repair genes show later median ages despite being high-risk genes. Pairwise gene-level comparisons using Mann–Whitney U tests with Benjamini–Hochberg FDR correction confirmed significant differences in median age between *BRCA1* and *PALB2* (q = 3.77 × 10^−3^) and between *TP53* and *PALB2*, *MSH6*, and *PMS2* (all q ≤ 0.019), supporting that not all high-penetrance genes are associated with equally early disease onset (Figure 6). These findings suggest that within this referral-based cohort, hereditary cancer susceptibility is not confined to very early-onset disease.

Age-stratified gene-specific analyses also revealed a clearer enrichment of *BRCA1* than *BRCA2* among younger patients. In the overall cohort, *BRCA1* P/LP findings were most frequent in patients diagnosed before age 40 (152/1867; 8.14%), compared with 59/1867 (3.16%) for *BRCA2*. A similar pattern was observed in breast cancer, where *BRCA1* prevalence was 8.01% in patients aged <40 years versus 3.21% for *BRCA2*, with both rates decreasing in older age groups and reaching a minimum of 1.98% each in patients aged ≥70 years. In ovarian cancer, *BRCA1* remained the predominant high-penetrance gene across most age bands, peaking at 15.52% in patients aged 40–49 years and remaining at 13.65% at ages 50–59, with a minimum of 3.25% in the ≥70-year group, whereas *BRCA2* frequencies were lower throughout (2.50–5.22%). Consistent with this age-group pattern, the median age at diagnosis was lower for *BRCA1* than *BRCA2* carriers overall (43 vs. 45 years; Mann–Whitney *p* < 0.05 before FDR correction; q = 0.119 after FDR adjustment across all 45 high-penetrance gene pairs).

Next, the association between age at diagnosis and the presence of multiple P/LP findings within the same patient was explored. Multi-gene P/LP carriers were identified in 131 of 9084 patients (1.44% of the overall cohort; 7.27% of P/LP-positive patients), of whom 102 (77.9%) had breast cancer, 26 had ovarian or fallopian/peritoneal cancer, and only 2 had endometrial cancer. Multi-gene carriers did not show an earlier median age at diagnosis than single-gene carriers overall (45 vs. 45 years, *p* = 0.592) and were only marginally younger than P/LP-negative patients (45 vs. 46 years, *p* = 0.0505). In breast cancer, carriers of ≥2 distinct P/LP alterations had a median diagnosis age of 43 years, compared with 44 years in single-gene carriers and 45 years in P/LP-negative patients; the comparison with P/LP-negative patients was statistically significant (*p* = 0.00127), whereas the comparison with single-gene carriers was not (*p* = 0.103). In ovarian cancers, no significant differences in age at diagnosis were detected (54 vs. 54 vs. 57 years for multi-gene, single-gene, and negative patients, respectively; multi-gene vs. single-gene *p* = 0.971; multi-gene vs. negative *p* = 0.278), although the small number of multi-gene carriers in this subgroup (n = 26) limits statistical power. For the remaining tumor types, the limited number of multi-gene carriers precluded subgroup comparisons.

Another hypothesis examined was whether the presence of additional intermediate-risk A complementary exploratory analysis examined whether additional intermediate-risk or low/unclassified-risk gene alterations alongside a same-tier variant might be associated with earlier age at diagnosis. No significant age differences were observed in any of these comparisons. Given the small subgroup sizes and the predominance of breast cancer among multi-gene carriers, these analyses should be regarded as exploratory and hypothesis-generating. Larger and more balanced cohorts will be required for formal assessment.

### 3.4. Multivariable Analysis of Factors Associated with P/LP Findings

**Figure 7:**
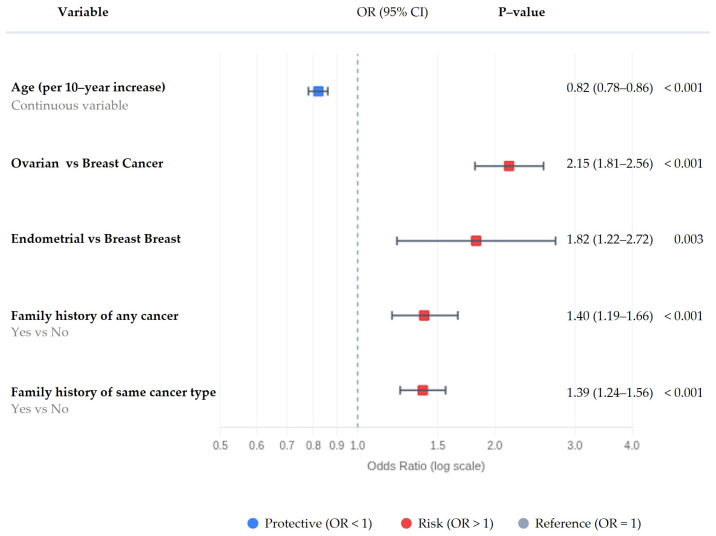
Forest plot showing adjusted odds ratios and 95% confidence intervals from the primary multivariable logistic regression model evaluating factors associated with detection of at least one pathogenic or likely pathogenic germline variant. Age was modeled continuously per 10-year increase.

In multivariable logistic regression analysis, adjusting for tumor type and family history, age at diagnosis remained independently associated with the likelihood of detecting a pathogenic or likely pathogenic (P/LP) variant. Specifically, each 10-year increase in age was associated with a 18% reduction in the odds of identifying a P/LP finding (OR = 0.82, 95% CI 0.78–0.86) (Figure 7).

To assess potential non-linearity in the age-P/LP relationship, we performed sensitivity analyses modeling age as categorical groups (<40, 40–49, 50–59, 60–69, ≥70 years). Using age <40 years as reference, adjusted odds ratios showed a consistent stepwise decline: 40–49 years OR 0.71 (95% CI 0.62–0.81), 50–59 years OR 0.64 (0.55–0.76), 60–69 years OR 0.53 (0.43–0.66), and ≥70 years OR 0.45 (0.34–0.60), and all comparisons remained statistically significant after FDR correction. The categorical model did not significantly improve fit over the continuous linear model (likelihood ratio test *p* = 0.140), supporting the log-linear assumption for overall P/LP detection.

Tumor type was also significantly associated with hereditary positivity, with higher odds observed in ovarian-related cancers (OR = 2.15) and endometrial-related cancers (OR = 1.82) compared to breast cancer. In addition, both measures of family history were independently associated with increased likelihood of a positive result, including any cancer family history (OR = 1.40) and family history of the same cancer type (OR = 1.39).

Taken together, these findings indicate that younger age, tumor type, and family history each contribute independently to the probability of identifying clinically relevant germline variants.

When the analysis was restricted to high-risk P/LP variants, the association between age and hereditary positivity became more pronounced. Each 10-year increase in age was associated with a 28% reduction in the odds of detecting a high-risk variant (OR = 0.72, 95% CI 0.67–0.78). Categorical age analysis revealed evidence of non-linearity (likelihood ratio test *p* = 0.001), with adjusted ORs of 0.53 (95% CI 0.45–0.64) for age 40–49 years, 0.43 (0.34–0.53) for 50–59 years, 0.41 (0.31–0.54) for 60–69 years, and a particularly pronounced effect in the oldest age group (≥70 years OR 0.28, 95% CI 0.19–0.41, all *p* < 0.0001). This non-linear pattern, with a steeper decline in the oldest age group, reinforces that, within this referral-based cohort, younger patients showed higher frequencies of high-penetrance findings.

In this model, tumor type showed a stronger effect compared to the overall P/LP analysis, with ovarian-related cancers demonstrating markedly increased odds relative to breast cancer (OR = 4.07), followed by endometrial-related cancers (OR = 2.20). Family history was also more strongly associated with high-risk findings, including both any cancer family history (OR = 1.73) and same-cancer family history (OR = 1.88).

These results suggest that the age-related decline in hereditary positivity is primarily driven by high-penetrance variants with established clinical relevance.

In a sensitivity analysis including only age and tumor type, age remained significantly associated with P/LP positivity, with each 10-year increase corresponding to a reduction in odds (OR = 0.84, 95% CI 0.80–0.88). The consistency of this finding across models supports the robustness of the observed association between age and the detection of hereditary variants.

## 4. Discussion

In the present study, a large cohort of 9084 women with breast cancer and gynecological malignancies (ovarian, endometrial, and cervical) underwent multigene panel genetic testing based on referral from a treating physician (oncologists or surgeons) due to suspicion of cancer predisposition. Clinically actionable variants were detected in one-fifth of the patients tested in accordance with previous studies in our and other diagnostic laboratories [24,35]. Significant percentages of P/LP variants were identified in all tumor types: 19.24% in breast cancer, 27.59% in ovarian cancer, 26.67% in endometrial cancer, and 22.73% in cervical cancer patients. The subgroup findings for endometrial and especially cervical cancer should be considered cautiously, given the marked imbalance in tumor-group representation and the limited precision of estimates outside the breast and ovarian cohorts. Additionally, although the positivity rate appears higher in the ovarian, endometrial, and cervical cancer subgroups, it should be noted that these patients differed from breast cancer patients in age distribution and in eligibility criteria for referral. As a result, baseline enrichment for hereditary risk may have contributed to the observed tumor-type differences in yield.

Accurate NGS analysis is crucial for delivering reliable results and increasing the test’s diagnostic rate. All types of variants were identified, with truncating variants accounting for the majority of alterations (73.24%), which is in agreement with the known loss-of-function effect of cancer-related alterations, which is a known mechanism of oncogenesis [36]. Among those, CNVs represent an often challenging-to-identify alteration type that may be missed by NGS approaches not coupled with appropriate bioinformatic pipelines. Notably, CNVs account for a substantial proportion of clinically relevant variants, may be identified in over 1% of cases, and often involve high-penetrance genes such as *BRCA1/2* [37,38,39]. Therefore, their detection is essential for comprehensive hereditary cancer risk assessment.

In addition to P/LP variants, the analysis revealed that VUS was the highest-tier variant, detected in 42.21% of cases. However, this rate was significantly lower (15.37%) in high-risk, well-characterized genes with management guidelines such as *BRCA1/2*, MMR genes, and *PALB2* [40,41,42]. Importantly, unlike P/LP findings, VUS did not show a meaningful age-related enrichment pattern in our cohort, supporting the distinction between uncertain and clinically established hereditary findings. Current evidence indicates that VUS should not be used to guide clinical management, and medical decision-making should instead remain based on personal and family history, as well as established pathogenic findings. At the same time, VUS should be reported and undergo periodic review, because reinterpretation over time may lead to reclassification as new evidence accumulates [43]. Notably, in hereditary cancer testing, most reclassified VUS are ultimately downgraded to benign or likely benign categories [40,42,43,44].

Several previous studies have shown that hereditary test results may change patient management and therapeutic approaches upon diagnosis, including risk-reducing bilateral mastectomy and hysterectomy, in accordance with current clinical guidelines [14,15,45]. Germline testing also helps estimate the risk of tumor recurrence and informs prognosis, with studies showing that carriers of certain mutations (e.g., *BRCA1/2*) have higher risks of recurrence but may achieve better outcomes with timely preventive surgery [46]. Specific recommendations exist mainly for high-risk genes and genes of intermediate penetrance [9]. Importantly, the clinical actionability of P/LP findings is not uniform across all genes included in multigene panels. Some alterations have more immediate implications for treatment selection or surgical planning, particularly *BRCA1/2*, whereas others are primarily relevant to syndrome recognition (e.g., *PTEN*, *TP53*, and *STK11*), surveillance, risk-reduction strategies, and cascade testing in relatives, such as mismatch repair genes in the gynecologic setting or selected moderate-risk genes including *PALB2*, *BRIP1*, *RAD51C*, and *RAD51D* [9,29]. For several female tumors, the most commonly altered genes are *BRCA1/2*. However, as previously observed, a broader genetic landscape of gene alterations beyond *BRCA1/2* can be identified when the genetic analysis includes other cancer susceptibility genes. In our cohort, breast and ovarian cancers were predominated by *BRCA1/2* gene alterations (6.78% and 15.75% respectively), while endometrial cancer presented mainly alterations in classical Lynch syndrome-associated genes [47]. However, high-risk alterations beyond *BRCA1/2* were present in all tumor subgroups, indicating the utility of multigene analysis [5,10,48]. Nevertheless, it should also be noted that expanded gene panel testing has also disadvantages as it may introduce additional complexity to risk interpretation, particularly for moderate- and low-penetrance genes and higher rates of VUS [4,6]. Indeed, a VUS was the higher-tiered variant detected in 42.21% of the patients tested in our cohort.

Furthermore, germline pathogenic variants are increasingly recognized as important modifiers of tumor behavior, prognosis, and therapeutic response, in addition to their use in hereditary risk assessment. In comparison to non-carriers, *BRCA*-associated breast malignancies in younger women have been associated with more aggressive features, such as a higher grade, triple-negative subtype, and higher proliferative index, as well as with worse long-term oncologic outcome [49]. In addition, genetic analysis provides information for treatment decision-making, especially for *BRCA1/2* and MMR gene alterations, thereby increasing the importance of genetic testing. These therapeutic implications support broadening genetic testing eligibility for patients with cancer beyond the presence of family history alone [11]. Given the substantial information provided by such testing, the main concern remains the identification of the eligible cohort for genetic testing. Apart from family history, the age of diagnosis remains a key factor in determining physicians’ choice of referral for genetic testing.

Although the present study was limited to germline findings and did not include matched tumor or multi-omics data, the detected variants can be viewed within established precision oncology frameworks. Hereditary cancer predisposition genes identified in our cohort map to several biologically and clinically relevant pathways. In addition to homologous recombination repair (e.g., *BRCA1*, *BRCA2*, *PALB2*, *ATM*, *CHEK2*) and mismatch repair pathways (e.g., *MLH1*, *MSH2*, *MSH6*, *PMS2*, *EPCAM*), other important biologic programs represented included *TP53*-associated genome surveillance/cell-cycle control, *CDH1*-associated cell-adhesion pathways, *PTEN/STK11*-associated PI3K/AKT/mTOR signaling, and NF1-associated *RAS/MAPK* pathway dysregulation [50,51,52,53,54,55]. This pathway-level perspective supports the relevance of multigene testing beyond individual gene lists and aligns with contemporary approaches to personalized cancer risk assessment. Future studies integrating matched tumor sequencing and publicly available multi-omics resources may further clarify age-related molecular patterns and their relevance for personalized management.

Studies show that while the prevalence of pathogenic variants decreases with age, a meaningful proportion of elderly patients still carry actionable mutations that can influence treatment decisions and surgical management [56]. For example, a pivotal study published in 2020 showed that 3.55% of unselected postmenopausal breast cancer patients carried pathogenic genetic mutations, specifically in cancer-associated genes like *BRCA1/2*, *ATM*, *CHEK2*, and *PALB2*. This was notably higher than the 1.29% prevalence found in cancer-free women, a finding suggesting that the role of genetic testing may need to be expanded for older patients [56]. Universal testing programs have demonstrated higher detection rates of pathogenic variants than guideline-directed testing and have led to changes in clinical management for many out-of-criteria patients, regardless of age [10,57,58]. Although some data suggest limited benefits for universal testing in specific subtypes like triple-negative breast cancer diagnosed after age 60 without other risk factors, overall evidence favors broader testing to avoid missing P/LP alteration carriers [13]. However, prevalence data alone do not establish a sufficient basis for testing-policy recommendations, since cost-effectiveness, health system capacity, and cascade testing uptake are equally important determinants of whether broader testing strategies are feasible and beneficial in clinical practice. The integration of these dimensions into prospective real-world evaluations is needed before broader testing approaches can be endorsed at the policy level [10,59,60].

Our results, in accordance with previous studies, indicated that, as expected, the prevalence of P/LP findings was higher in younger patients and declined with age range, from 24.37% in those aged <40 years to 15.90% in those aged ≥70 years, an absolute difference of 8.47 percentage points [61,62]. In contrast, both negative and VUS results did not show a meaningful age-related pattern. The categorical age-group analyses provide a more interpretable expression of the age effect, with the reduction in P/LP detection concentrated in patients aged <40 years compared with all older groups, particularly for high-penetrance findings (absolute difference 6.76 percentage points). These results indicate that the age signal is driven by the younger end of the distribution rather than by a smooth gradient across the full age spectrum.

**Figure 8:**
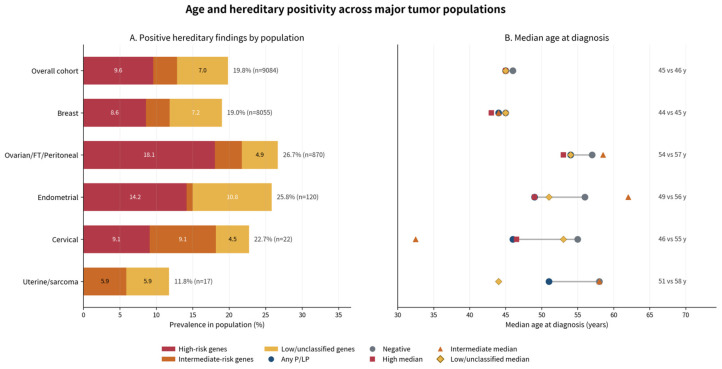
Age and hereditary positivity in the entire cohort and across major tumor types analyzed. (**A**) Distribution of positive hereditary findings across tumor types. Bars represent the percentage (%) of patients harboring P/LP variants, stratified by gene risk category: high-risk (red), intermediate-risk (orange), and low- or unclassified-risk genes (yellow). Total positivity rates and sample sizes (*n*) are indicated for each tumor group. (**B**) Median age at diagnosis according to variant classification. Symbols indicate median age for patients with high-risk (red squares), intermediate-risk (orange triangles), low/unclassified variants (yellow diamonds), and negative results (gray circles). Horizontal lines represent the range of median ages observed across groups where applicable. Overall median ages for each tumor type are shown on the right.

Importantly, clinically relevant findings were still identified among older patients within this referral-based cohort, despite the lower positivity rate. Across the overall cohort, the observed age-related signal appears to be driven predominantly by high-penetrance variants, which are most frequent in younger patients, decreasing from 13.87% in individuals aged <40 years to 7.11% in those aged ≥70 years, while a statistically significant difference can also be observed in the median age of diagnosis for all tumor types (Figure 8).

Importantly, gene-specific analyses further refine this interpretation by demonstrating that not all high-penetrance genes are associated with equally early disease onset. Among high-risk P/LP variants, alterations in *TP53*, *PTEN*, and *BRCA1* were associated with the youngest median ages at diagnosis, whereas *PALB2* and mismatch repair genes presented at relatively older ages despite their high penetrance. This highlights the heterogeneity within the high-risk category and reinforces that hereditary cancer susceptibility extends beyond strictly early-onset disease [61,63]. In addition, a clear age-dependent enrichment of *BRCA1* compared to *BRCA2* was observed. In the overall cohort, *BRCA1* P/LP variants were most frequent in patients diagnosed before age 40 (8.14%) compared with BRCA2 (3.16%), a pattern mirrored in breast cancer and persisting across age groups, although both genes showed declining frequencies with increasing age. In ovarian cancer, *BRCA1* remained the predominant high-risk gene across most age groups, with consistently higher frequencies than *BRCA2*. This difference is further supported by the younger median age at diagnosis observed in *BRCA1* compared to *BRCA2* carriers (43 vs. 45 years), underscoring distinct biological and clinical behaviors even among closely related high-penetrance genes.

Even though younger patients are more likely to have high-penetrance variants, a substantial number of clinically useful findings are still present in all age groups, even those 70 years or older. It is important to note that high-risk variants can still be found in older patients at a rate that is not negligible, and that intermediate- and low-penetrance variants have distributions that are relatively stable across all age groups. Moreover, the finding that specific high-penetrance genes, including *PALB2* and mismatch repair genes, correlate with later ages at diagnosis highlights that hereditary cancer predisposition is not limited to early-onset disease. These results suggest that restricting genetic testing solely based on age could lead to underdiagnosis of inherited predisposition-associated findings in elderly individuals who might still benefit from tailored surveillance, treatment options, and cascade testing of potentially affected family members [64,65]. Therefore, more inclusive testing approaches that extend beyond conventional age-related guidelines warrant consideration, especially concerning multigene panel testing.

The present study represents a large real-world cohort of women undergoing multigene hereditary cancer testing, predominantly composed of patients with breast cancer, with additional representation of gynecologic malignancies. Within this context, age at diagnosis was independently associated with the likelihood of detecting germline P/LP variants, even after adjustment for tumor type and family history. Specifically, each 10-year increase in age was associated with an approximately 18% reduction in the odds of identifying a P/LP finding (OR 0.82, 95% CI 0.78–0.86), indicating a gradual decline in hereditary positivity with increasing age. Consistent with this, age-group comparisons showed modest absolute differences in P/LP prevalence across categories. Sensitivity analyses using categorical age groups demonstrated a consistent stepwise decline in P/LP detection with increasing age, supporting the use of a linear model for the primary analysis.

When stratified by variant category, this association was more pronounced for high-risk variants, for which each 10-year increase in age was associated with an approximately 28% reduction in odds (OR 0.72, 95% CI 0.67–0.78). This suggests that the age-related pattern is driven primarily by high-penetrance variants, although the overall magnitude of the effect remains moderate. Given the predominance of breast cancer cases in the cohort, this finding largely reflects patterns within breast cancer, while remaining directionally consistent across ovarian and endometrial cancers. The higher odds of P/LP findings observed in ovarian cancer are in line with established evidence supporting a stronger hereditary component in this tumor type. However, these findings should be interpreted in the context of the smaller sample size of gynecologic cancers compared to breast cancer within this cohort.

Age and family history operate as complementary predictors of hereditary cancer risk in our cohort. In addition, current guideline-based eligibility testing criteria incorporate both age and family history and likely capture a substantial proportion of older patients with hereditary findings, particularly those with strong personal or family histories suggestive of hereditary predisposition. Our findings, therefore, do not establish a deficiency of age-based eligibility rules in isolation, but rather indicate that, within a referral-based cohort, clinically relevant hereditary findings are not confined to the youngest patients.

### Strengths and Limitations

This study benefits from a large cohort size of 9084 patients with breast and gynecological malignancies undergoing multigene panel testing, providing robust statistical power and enabling comprehensive evaluation across tumor types.

Additionally, the study offers comprehensive age-stratified analyses, enriching current knowledge of the distribution of P/LP variants across age groups and showing that clinically significant findings extend beyond early-onset disease. The gene-specific assessment emphasizes the variability of hereditary cancer risk, thereby enhancing the clinical significance of the results.

Several limitations should also be acknowledged. The main limitation of the study is its retrospective design, and the dependency on a referral-based cohort may have introduced selection bias, as patient selection for genetic testing may have been influenced by clinical indicators such as age at diagnosis or the presumption of hereditary risk. This selection bias limits the generalizability of our findings and precludes direct conclusions regarding testing strategies in unselected populations. Accordingly, conclusions interpreting the potential value of age-unrestricted or universal testing should be interpreted with caution. Our results support the observation that clinically actionable variants can be identified across all age groups within a referred population, but do not, by themselves, establish the benefit of universal testing in the broader cancer population. Moreover, the gene penetrance categorizations used reflect current consensus and clinical relevance to the tumor types analyzed, rather than fixed penetrance estimates, and may evolve as new evidence accumulates.

An additional limitation is the large sample size, which increases statistical power to detect small differences. Some statistically significant comparisons may therefore reflect modest effect sizes with limited clinical relevance, and our findings should be interpreted alongside effect estimates and confidence intervals rather than *p*-values alone. Moreover, the multivariable models included only a limited set of covariates—age at diagnosis, tumor type, and binary family-history indicators. Several potentially relevant confounders, including ancestry, tumor receptor status, specific clinical indications for genetic testing referral, and other tumor-specific clinicopathologic features, were not available and could not be incorporated. Although the cohort is predominantly of Greek ancestry, structured individual-level ancestry data were not systematically captured, limiting interpretation of the observed VUS rate and of gene-specific penetrance assumptions in this population.

Furthermore, detailed family history data were absent for many patients, constraining the evaluation of the impact of family history on variant prevalence. Consequently, a documented assessment of the existing guideline-based testing criteria was not possible. Family history was captured only as binary indicators (any cancer; same cancer), without information on the number, degree, or age at diagnosis of affected relatives or bilateral disease, which may have led to incomplete adjustment for familial risk and limits the robustness of the independent age effect. Furthermore, clinical follow-up data were missing, preventing the evaluation of the direct impact of genetic findings on patient outcomes, treatment decisions, or survival. Finally, cost-effectiveness, health system capacity, and cascade testing uptake, all of which are essential for evaluating the feasibility and benefit of broader testing strategies, were not addressed; prospective evaluations linking diagnostic yield to these factors will be required before broader testing approaches can be endorsed at the policy level.

## 5. Conclusions

In conclusion, this large cohort study indicates that NGS-based genetic testing using a panel of cancer-associated genes offers significant insights for patients with breast and gynecological tumors. Approximately one in five patients had genetic alterations linked to hereditary cancer predisposition. Within this referral-based cohort, the prevalence of high-penetrance variants was higher in younger patients; nonetheless, clinically relevant, actionable findings were identified across all age groups, including older patients referred for testing. These results suggest that reliance on age alone as a criterion for genetic testing in patients with cancer may be insufficient, and that broader testing approaches could improve the identification of hereditary cancer risk and inform patient management, although further evaluation in unselected populations is warranted.

## Figures and Tables

**Table 1:** Tumor types analyzed in the cohort and median age of diagnosis.

Characteristic	Total *n*	Median Age at Diagnosis, Years (IQR)
Full cohort	9084	45 (40–53)
Breast cancer	8055	45 (40–52)
Ovarian Cancer	870	57 (48–67)
Endometrial cancer	137	55 (49–62)
Cervical cancer	22	49 (45–60)

**Table 2:** Age-stratified distribution of genetic testing outcomes (Negative, VUS, and P/LP Variants).

Age Group, Years	Negative *n* (%)	VUS *n* (%)	P/LP *n* (%)	Total *n*
<40	35.14%	40.49%	24.37%	1867
40–49	37.27%	43.48%	19.25%	4025
50–59	38.37%	41.79%	19.85%	1814
60–69	41.44%	40.89%	17.67%	900
≥70	41.84%	42.26%	15.90%	478
Overall comparison across age groups				χ^2^(8) = 37.77; *p* = 8.31 × 10^−6^
Negative vs. VUS				*p* = 0.201
Negative vs. P/LP				*p* = 2.53 × 10^−6^
VUS vs. P/LP				*p* = 1.51 × 10^−4^

**Table 3:** Age-stratified distribution of P/LP variants by gene risk category.

Age Group	*n*	High *n* (%)	Intermediate *n* (%)	Low/Unclassified *n* (%)	Any P/LP *n* (%)
<40	1867	13.87%	2.20%	8.30%	24.37%
40–49	4025	8.57%	2.46%	8.22%	19.25%
50–59	1814	8.60%	2.37%	8.88%	19.85%
60–69	900	9.22%	3.00%	5.44%	17.67%
≥70	478	7.11%	2.72%	6.07%	15.90%

## Data Availability

The sequencing data (FASTQ files) generated in this study are not publicly available due to the presence of potentially identifiable human genomic information and restrictions imposed by GDPR and the study’s ethical approvals. Access to the raw data may be granted upon reasonable request and subject to approval by the relevant ethics committee. Processed data are provided in the article and Supplementary Materials.

## Supplementary Materials

The following supporting information can be downloaded at: https://www.mdpi.com/article/10.3390/cancers18101541/s1, Table S1: Patient-level summary of germline genetic testing in the breast and gynecologic cancer cohort (n = 9084). Table S2: Detailed variant-level annotations of pathogenic, likely pathogenic, VUS, CNV, and recessive findings detected in the cohort.

## Abbreviations

The following abbreviations are used in this manuscript:

P/LP	Pathogenic/Likely Pathogenic
NGS	Next Generation Sequencing
OR	Odds Ratio
VUS	Variants of Uncertain Significance
CNV	Copy Number Variation
